# Supplementary material for: Short-stay urgent hospital admissions of children with convulsions: A mixed methods exploratory study to inform out of hospital care pathways
Source: PLoS One. 2024 Apr 1;19(4):e0301071. doi: 10.1371/journal.pone.0301071 (PMC10984513; doi:10.1371/journal.pone.0301071)
Supplement: S1 File — (DOCX) [file pone.0301071.s001.docx]

**The FLAMINGO project (FL**ow of **A**d**M**issions in ch**I**ldren and you**NG** pe**O**ple)

**Health Professionals - Topic Guide on Convulsions**

Can you start by confirming what your role is and your employer?

Probes – confirm role, employer, how long they have been in this role.

Can you tell me about the seizure service in [Healthboard]? Can you describe the care pathway? Probes: Number of staff, 24 hr or 9-5, where staff are based. Are they aware of differences between other health boards?

**Experience of short stay admissions (SSAs)**

We are interested in unplanned or emergency hospital admissions in children, particularly short stay admissions (SSAs) where a child is admitted and then discharged from hospital on the same day (less than 24 hours)

Can you tell me about your experiences of SSAs of children?

**For convulsions in the community care pathway**

Prompt interviewee to think about the differences in care pathways and potential interventions to improve care for the following scenarios:

| **Convulsion Type** | **Previously well child** | **Long term conditions/ACEs** |
| --- | --- | --- |
| First non-febrile |  |  |
| Recurrent non- febrile |  |  |
| First febrile |  |  |
| Recurrent febrile |  |  |

Use table above to prompt the different scenarios and to what extent one size fits all for convulsions in the community or whether there are/ could be different care pathways.

Have you had experience (may be personal - family - which is OK) of any other hospital (s) / emergency services for children in Scotland? Can you give me a specific examples of how care pathways can be different?

Can you tell me about your experience of receiving referrals? Probe: How easy is to distinguish between febrile and afebrile convulsions? What is the difference between children with a history of convulsions vs first convulsions?

Does the service differ for children with long term conditions vs children who are generally well? Probe: Are children with long term conditions looked after elsewhere

Parental anxiety and competence – what impact does this have and how does it vary between first convulsion and families who are more experienced? What role do the child and family’s circumstances have in the decision-making process, if any?

Do you think that any hospital admissions of less than 24 hours could be safely avoided?

Probe – how?

Some people we have talked to refer to appropriate and inappropriate admissions. What are your thoughts about these terms….? What do you consider to be an appropriate referral of an unwell child to a hospital for a SSA? Tell me about some of your experiences.

**Readmissions**

Is there any support available to parents when their child has been discharged home?

What are the issues with convulsions happening in the community?

Tell me about your experiences of readmissions. [Focus on readmissions, not on continuing care]

Probes: is this something that happens frequently/infrequently; when these types of readmissions do occur, what do you think are the reasons; factors which contribute to this; typical presentation – child/family)

**Communication processes between the professional referring and the professional admitting child to hospital**

Can you think of an example where communication, between the professional referring and the professional admitting child to hospital, went really well?

- - What helped?

Can you think of an example where communication, between the professional referring and the professional admitting child to hospital, did not run so smoothly?

- - What were the issues, what were the consequences, how could this be improved?

What role do specialist epilepsy nurses have compared to paediatric A&E nurses? How does this change depending on the hospital – e.g. district hospital vs children’s hospital.

**Current situation – COVID**

With the current situation with COVID-19 are you seeing a change in children attending hospital for unplanned visits?

How do you think care of sick children at home is changing as a result of Covid19?  Prompts… are there any particular examples you have come across?  Tell me more…. Any different examples…?

Are there any changes that you think will or should remain after the COVID period? [E.g. telehealth etc.]

What is the role of video technology? E.g. videoconferencing or videoing convulsions.

**Final questions- looking forward**

Thinking about moving forward and SSAs in children….

- What would be your recommendations for change regarding an acute convulsion in the community(?)

Probes- Seizure grid scenarios. change in the short/medium/ longer term? Who, where, in what circumstances, training? Is there a potential role for VC and technology in preventing SSAs

- Convulsions are in top 10 for SSAs, all the others are infectious. Do you think that all SSAs should follow same pathway? Potentially follow on from COVID changes to hospitals.
- Are there any particular issues or health conditions you’d highlight for the next stages of our research?
- What do you think will be happening with children’s SSAs in 5 years’ time?
