# Supplementary material for: Short-stay urgent hospital admissions of children with convulsions: A mixed methods exploratory study to inform out of hospital care pathways
Source: PLoS One. 2024 Apr 1;19(4):e0301071. doi: 10.1371/journal.pone.0301071 (PMC10984513; doi:10.1371/journal.pone.0301071)
Supplement: S2 File — (DOCX) [file pone.0301071.s002.docx]

C719 malignant neoplasm

F840 Autism

G403 Generalised Epilepsy

G408 Other Epilepsy

G409 Epilepsy

G801 Spastic Diplegia

G802 Spastic Hemiplegia Cerebral Palsy

G803 Dyskinetic Cerebral Palsy

G808 Other Cerebral Palsy

G809 Cerebral Palsy

G819 Hemiplegia

P072 Extreme Immaturity

Q850 Neurofibromatosis

Q851 Tuberos Sclerosis

R620 Delayed milestones
