# Supplementary material for: Short-stay urgent hospital admissions of children with convulsions: A mixed methods exploratory study to inform out of hospital care pathways
Source: PLoS One. 2024 Apr 1;19(4):e0301071. doi: 10.1371/journal.pone.0301071 (PMC10984513; doi:10.1371/journal.pone.0301071)
Supplement: Attachment — Submitted filename: PONE-D-23-34920_Response to reviewers.docx [file pone.0301071.s003.docx]

## **Manuscript PONE-D-23-34920 - Authors’ responses to reviewer comments:**

Any changes or amendments the authors have made to the manuscript in line with reviewer comments can be identified by the use of yellow highlighting.

### **Reviewer 1 Comments and Author Responses**

Thank you for your insightful and helpful comments on our manuscript. We have considered them in depth and would like to offer the following responses to each of the comments and suggestions proposed:

**Comment 1** – Specify with more details the contribution of your work with respect to the previous ones published by you. As you stated, “The main FLAMINGO study findings are published elsewhere [13-14]”, and the contribution of this work should be emphasised better.

**Author Response**

On page 3 we have described the focus of the publications reporting the main FLAMINGO study findings (new text in yellow):

‘The main FLAMINGO study findings, published elsewhere, report on quantitative data linkage analysis of routinely acquired Scottish data to identify referral sources and characteristics for all urgent paediatric SSAs [13] and on qualitative interviews with 21 parents and 48 health professionals communicating their desired outcomes of pre-hospital urgent care for all causes of SSAs [14].

**Comment 2** – Suggest what might be the future direction of your studies. For instance, what about the adoption of AI to support the stratification of your patients? This is a sector in which the literature (and the medicine) is going, and it might be interesting to highlight this opportunity (eg. See Falavigna et al., 2019; Ippoliti et al., 2021).

Citation:

Falavigna, G., Costantino, G., Furlan, R., Quinn, J. V., Ungar, A., & Ippoliti, R. (2019). Artificial neural networks and risk stratification in emergency departments. Internal and Emergency Medicine, 14, 291-299.
Ippoliti, R., Falavigna, G., Zanelli, C., Bellini, R., & Numico, G. (2021). Neural networks and hospital length of stay: an application to support healthcare management with national benchmarks and thresholds. Cost Effectiveness and Resource Allocation, 19(1), 1-20.

**Author Response** –

Thank you for suggesting that we could indicate areas for future research. We have added suggestions on pages 25 and 26 as follows:

‘Future research is warranted to verify the impact of open access to hospital wards for children with known convulsions on SSAs.’

‘Future research would be of merit to investigate the impact of implementation of guidelines and interventions on SSAs for convulsions.’

We enjoyed reading and discussing these two papers (Falavigna et al. 2019; Ippoliti et al. 2021) and agree there is potential in adopting AI to support the stratification of patients within the ED and manage patient flow. It has relevance to our wider FLAMINGO project, and we will refer to back these citations when writing future publications. However, with regard to this paper, the value of adopting a mixed method approach as we have done in this study, highlights the potential to intervene and provide care to children with convulsions *before* they arrive at the ED. Our findings support the consideration of pre-hospital pathways for paediatric convulsions and therefore the discussion around potential use of AI is not relevant here.

We have also taken the opportunity to correct minor typographical errors or omissions indicated in yellow highlighter throughout.

Yours faithfully,

Cari Malcom on behalf of the FLAMINGO team
