# Supplementary material for: Short-stay urgent hospital admissions of children with convulsions: A mixed methods exploratory study to inform out of hospital care pathways
Source: PLoS One. 2024 Apr 1;19(4):e0301071. doi: 10.1371/journal.pone.0301071 (PMC10984513; doi:10.1371/journal.pone.0301071)
Supplement: Attachment — Submitted filename: PONE-D-23-34920_Response to reviewers.docx [file pone.0301071.s004.docx]

## **Manuscript PONE-D-23-34920 - Authors’ responses to reviewer comments:**

### **Reviewer 1 Comments and Author Responses**

Thank you for your insightful and helpful comments on our manuscript. We have considered them in depth and would like to offer the following response:

**Comment 1** – I believe this article did not consider the main comment suggested in the first round of this review, which is fundamental to satisfy the key criteria of this journal, ie., whether the study presented the results of original research (i), and whether results reported have not been published elsewhere (ii). Indeed, authors do not explain properly how this study differs from the previous ones (published by authors on the same study), and they do not specify the contribution of this work with respect to the current (published) knowledge.

**Author Response**

The authors regret that the reviewer’s substantive comments were not appropriately addressed and are grateful for the opportunity to respond to them in this revised version. We are confident this manuscript meets the essential PLOS ONE criteria, that being the communication of original research that has not been previously published in the peer-reviewed literature. We hope that the justification and amendments below clearly articulate this.

The **Fl**ow of **A**d**m**issions in Ch**i**ldren and You**ng** People (FLAMINGO) project was designed to explore urgent short stay hospital admissions for acutely unwell children, namely their characteristics and referral patterns. Analysis of this data drew our attention to convulsions as a focus of interest given their higher prevalence in Emergency Department (ED) attendance and referral in comparison to the nine other clinical presentations included in the data. This informed our decision to undertake the following novel analyses: (i) stratification of quantitative data by febrile and afebrile convulsions, also presenting time of admission and details of readmissions (ii) further qualitative interviews with parents, professionals, including Epilepsy Specialist Nurses was undertaken to explore their experiences of Short Stay Admissions (SSAs) for children with afebrile and febrile convulsions (iii) thematic analysis of interviews. Our present paper arose from observations made in an earlier paper and, as explained above, presents novel results.

The novel contributions of this work with respect to the existing and published evidence base is communicated in detail within the discussion section, particularly highlighting the value of using a mixed methods approach which facilitates a better understanding of the complexities of care pathways leading to SSAs for children experiencing convulsions.

The results reported in this paper have not been published elsewhere. Below is a list of each FLAMINGO paper and a summary of any convulsion data published in them:

**Dick S, Kyle R, Wilson P et al. Insights from and limitations of data linkage studies: analysis of short-stay urgent admission referral source from routinely collected Scottish data. Archives of Disease in Childhood. 2023 Apr 1; 108(4): 300-6.** Convulsions are mentioned nine times, in context of the referral source in comparison to children presenting with other conditions. There is no stratification or presentation of data by febrile of afebrile status, readmission rates or time of admission.

**King E, Dick S, Hoddinott P et al. Regional variations in short stay urgent paediatric hospital admissions: a sequential mixed-methods approach exploring differences through data linkage and qualitative interviews. BMJ Open. 2023 Sep 1; 13(9): e072734.** Convulsions are referred to three times only in the context of differences in SSA rates between regions.

**Malcolm C, King E, France E, et al. Short stay hospital admissions for an acutely unwell child: a qualitative study of outcomes that matter to parents and professionals. PLOS ONE. 2022 Dec 16; 17(12): e0278777.** The is no reference to convulsions in this paper.

**King E, France E, Malcolm C, Kumar S, Dick S, Kyle RG, Wilson P, Aucott L, Turner S, Hoddinott P. Identifying and prioritising future interventions with stakeholders to improve paediatric urgent care pathways in Scotland, UK: a mixed methods study. BMJ Open. 2023 Oct 1; 13(10):e074141.** Convulsions are mentioned in the context of identifying convulsion as a presentation with a potential pathway of care – there is no convulsion data or findings presented or discussed.

Changes to manuscript

Any changes or amendments the authors have made to the manuscript in line with reviewer comments can be identified by the use of yellow highlighting:

The **Fl**ow of **A**d**m**issions in Ch**i**ldren and You**ng** Pe**o**ple (FLAMINGO) project was undertaken in response to the growing number of paediatric urgent hospital admissions in the UK [4-11], explained largely by rising SSAs [4,12]. This exploratory sequential mixed-methods study aimed to improve understanding of SSAs in Scotland through linkage of national databases to identify referral pathways and characteristics of SSAs. Qualitative interviews with professionals were complemented by parent experiences and Public Patient Involvement. The main FLAMINGO study findings, published elsewhere, report on quantitative data linkage analysis of routinely acquired Scottish data to identify referral sources and characteristics for urgent paediatric SSAs arising from a range of common conditions including, but not limited to, viral illness, asthma and bronchiolitis [13-14] and on qualitative interviews with 21 parents and 48 health professionals communicating their desired outcomes of pre-hospital urgent care for these urgent SSAs [15]. Data linkage findings from the main FLAMINGO dataset indicated a high prevalence of SSAs for convulsions presenting at the emergency department (ED) [13] which, given the lack of recently published evidence in this area, warranted further detailed investigation. Previous FLAMINGO papers [13-16] did not specifically report the characteristics of SSAs related to convulsions or parental and professional experiences of these admissions. Therefore, additional quantitative analysis and qualitative research involving the recruitment of Epilepsy Specialist Nurses (ESN)s was undertaken. This paper communicates novel findings from this research to identify learning for improvement in care pathways and development of potential interventions focused on safely reducing urgent SSAs for convulsions and improving care pathways, by analysing (i) characteristics of SSAs for children with afebrile or febrile convulsions, and (ii) qualitative interviews with professionals and parents with experience of childhood convulsions.
